# Approaching COVID‐19 with epidemiological genomic surveillance and the sustainability of biodiversity informatics in Africa

**DOI:** 10.1002/jmv.28308

**Published:** 2022-11-21

**Authors:** Abdullahi Tunde Aborode, Helen Huang, Andrew Awuah Wireko, Aashna Mehta, Jacob Kalmanovich, Toufik Abdul‐Rahman, Vladyslav Sikora, Aeshah A. Awaji

**Affiliations:** ^1^ Healthy Africans Platform Research and Development Ibadan Nigeria; ^2^ Mississippi State University Starkville Mississippi USA; ^3^ Royal College of Surgeons in Ireland University of Medicine and Health Sciences Dublin Ireland; ^4^ Sumy State University Sumy Ukraine; ^5^ University of Debrecen‐Faculty of Medicine Debrecen Hungary; ^6^ Drexel University College of Medicine Philadelphia Pennsylvania USA; ^7^ Department of Biology, Faculty of Science, University College of Taymaa University of Tabuk Tabuk Saudi Arabia

**Keywords:** Africa, biodiversity, bioinformatics, COVID variants, COVID‐19 pandemic, genomic surveillance, phylogenetics, SARS‐CoV‐2 infection

## Abstract

COVID‐19 is an acute respiratory illness caused by Severe Acute Respiratory Syndrome‐Coronavirus 2 (SARS‐CoV‐2). The first case was reported in Africa on February 14, 2020 and has surged to 11 million as of July 2022, with 43% and 30% of cases in Southern and Northern Africa. Current epidemiological data demonstrate heterogeneity in transmission and patient outcomes in Africa. However, the burden of infectious diseases such as malaria creates a significant burden on public health resources that are dedicated to COVID‐19 surveillance, testing, and vaccination access. Several control measures, such as the SHEF2 model, encompassed Africa's most effective preventive measure. With the help of international collaborations and partnerships, Africa's pandemic preparedness employs effective risk‐management strategies to monitor patients at home and build the financial capacity and human resources needed to combat COVID‐19 transmission. However, the lack of safe sanitation and inaccessible drinking water, coupled with the financial consequences of lockdowns, makes it challenging to prevent the transmission and contraction of COVID‐19. The overwhelming burden on contact tracers due to an already strained healthcare system will hurt epidemiological tracing and swift counter‐measures. With the rise in variants, African countries must adopt genomic surveillance and prioritize funding for biodiversity informatics.

## 1 INTRODUCTION

COVID‐19 is an acute respiratory illness caused by Severe Acute Respiratory Syndrome‐Coronavirus 2 (SARS‐CoV‐2). Initially reported in Wuhan, China, it was soon declared a pandemic by the World Health Organization (WHO) in March 2020.^1^

First reported in Africa on  February 14, 2020, approximately 11 million cases and 254 807 deaths have been observed as of July 2022, amounting to 2% of global cases.^2^ Of these, 43% and 30% of cases are reported from Southern and Northern Africa, respectively. South Africa, Morocco, Tunisia, Ethiopia, and Egypt account for more than half of the total confirmed COVID‐19 cases in Africa, with South Africa leading in terms of reported cases (4 million) and deaths (101 900).^1^, ^3^

Current epidemiological data demonstrate heterogeneity in transmission and patient outcomes in Africa, the cause of which remains unknown. Differences in times of introduction, health infrastructure, testing, surveillance, and vaccination access are various factors that could contribute to epidemiological differences, hindering a uniform public health response.^3^ Unique to Africa is the burden of other infections, such as malaria, which could present similarly and lead to misdiagnosis, especially if solely relying on clinical symptoms.^4^ Moreover, tackling multiple infectious diseases can significantly burden public health resources dedicated to COVID‐19. Other aspects such as socioeconomic status and health literacy can significantly impact adherence to public health policies.^3^ Determining the relationship between these factors can provide valuable insight into the burden of viral diseases and help mitigate policy formulation and implementation shortcomings.

## 2 PROSPECTIVE STRATEGIES USED TO CONTROL THE TRANSMISSION OF SARS‐COV‐2

Several control measures were implemented to reduce the spread of COVID‐19. The first few cases in Africa were introduced through foreign travel, with case fatality rates significantly lower (approximately 1.3%) than in European countries (13.4%). It was assumed that a younger population contributed to possibly lower mortality in the region. ^5^ As a result, prospective strategies have to be implemented to ensure that the scope of preventive policies was best representative of the current demographics. Initial measures included hand hygiene, protective equipment (masks, gowns), regular testing, contact tracing, and quarantine for infected and suspected cases.^5^ The Social distancing, Hand washing, Elbow, Face, and Feel (SHEF2) model can be described as a framework encompassing the most effective preventive measures.^6^ Physical distancing is most effective in curbing disease as person‐to‐person transmission severely affects up to 20% of people.^6^ Africa's pandemic preparedness has reached milestones in implementing comprehensive social measures. With the help of collaborations and international partnerships, healthcare workers were trained to employ effective risk‐management strategies in hospital settings and monitor patients in home settings.^7^ By reinforcing its capacities by deploying community health workers and receiving financial resources for essential patient monitoring such as oxygen concentrators, Africa has undoubtedly gained international momentum.

## 3 LIMITATIONS IN CURRENT STRATEGIES AND GAPS IN RESEARCH PREVENTING THE CONTROL OF COVID‐19 OUTBREAKS

Despite the implemented SHEF2 framework, hand washing and remaining at home while symptomatic continue to be challenging in communities. Lack of safe sanitation continues to be one of the most common causes of developing food‐borne illnesses and poses a serious risk amidst the COVID‐19 pandemic. Inaccessible drinking water from safe sources exacerbates the risk of developing viral illnesses and could lead to unnecessary fatalities, as seen in the Ebola virus epidemic. There is a significant need for personal protective equipment and related resources with a stable public health infrastructure to support COVID‐related strategies, both of which are major limiting factors for developing nations.^6^

The enforcement of travel restrictions and lockdowns were robust but likely posed detrimental consequences on the economy and the livelihood of people in Africa.^6^ Most people in Africa rely on daily income from multiple jobs, which makes it difficult to formulate and create adherent policies requiring homestay and social isolation.^5^, ^7^ In addition to these, current COVID‐19 restrictions fail to prevent outbreaks due to the lack of health infrastructure and unstable socioeconomic environment in African communities. As a result, there continues to be a lack of future direction to improve pandemic preparedness.

The cases of COVID‐19 in Africa continue to rise and are attributed to the lack of solutions on effective counter‐measures to prevent an outbreak, such as poor epidemiological tracing due to the overwhelming burden on contact tracers.^8^ The high transmissibility of variant strains, little information is known about the severity of these mutations as it accumulates and the efficacy of currently deployed vaccines towards new variants (Omicron and Delta).^9^, ^10^, ^11^, ^12^

Though preliminary studies suggest that the severity of mutations of the new COVID‐19 variants revealed higher case incidences in Omicron variants, studies in Africa have now uncoupled from the prevalence of severe disease due to the emergence of cell‐mediated immunity from past natural infection and vaccination (hospitalizations and deaths). Despite indications that the omicron variation avoids neutralizing antibody activity caused by spike‐protein‐based vaccinations and by earlier infection with other variants that did not carry the same entire set of supposedly antibody‐evasive mutations, a detachment has occurred.^13^ Studies also suggest omicron variants and beta variants are more capable of eluding neutralizing antibody activity.^14^, ^15^

Given the lack of information surrounding these variants, a resurgence in these strains amidst low‐resource settings and already strained healthcare centers will deteriorate the capacity and resources to control the outbreak. In addition, insufficient information, low healthcare response, low surveillance, and a lack of sustainable strategies to put these variants in order in Africa can endanger the public and weaken the healthcare system.^16^, ^17^, ^18^ Without proper diagnostic technologies, early detection of COVID‐19 and variants will be near to impossible and can mirror the failure in containing the Ebola virus outbreak in 2014.^19^, ^20^ Though western hemispheres have developed robust genomic laboratories that can curtail the surge of variants and its detection, there is a lack of research that suggests these actions would be upheld with the current state of Africa's national capacity. Due to that gap in research, no formulated plan took the specific conditions in Africa into account.^21^ Additional research must address these limitations and gaps unique to developing nations to improve COVID‐19 prevention and control in these regions.

## 4 THE EVOLUTION OF EPIDEMIOLOGICAL GENOMIC SURVEILLANCE AND ITS APPLICATIONS TO THE COVID‐19 PANDEMIC

**Figure 1:**
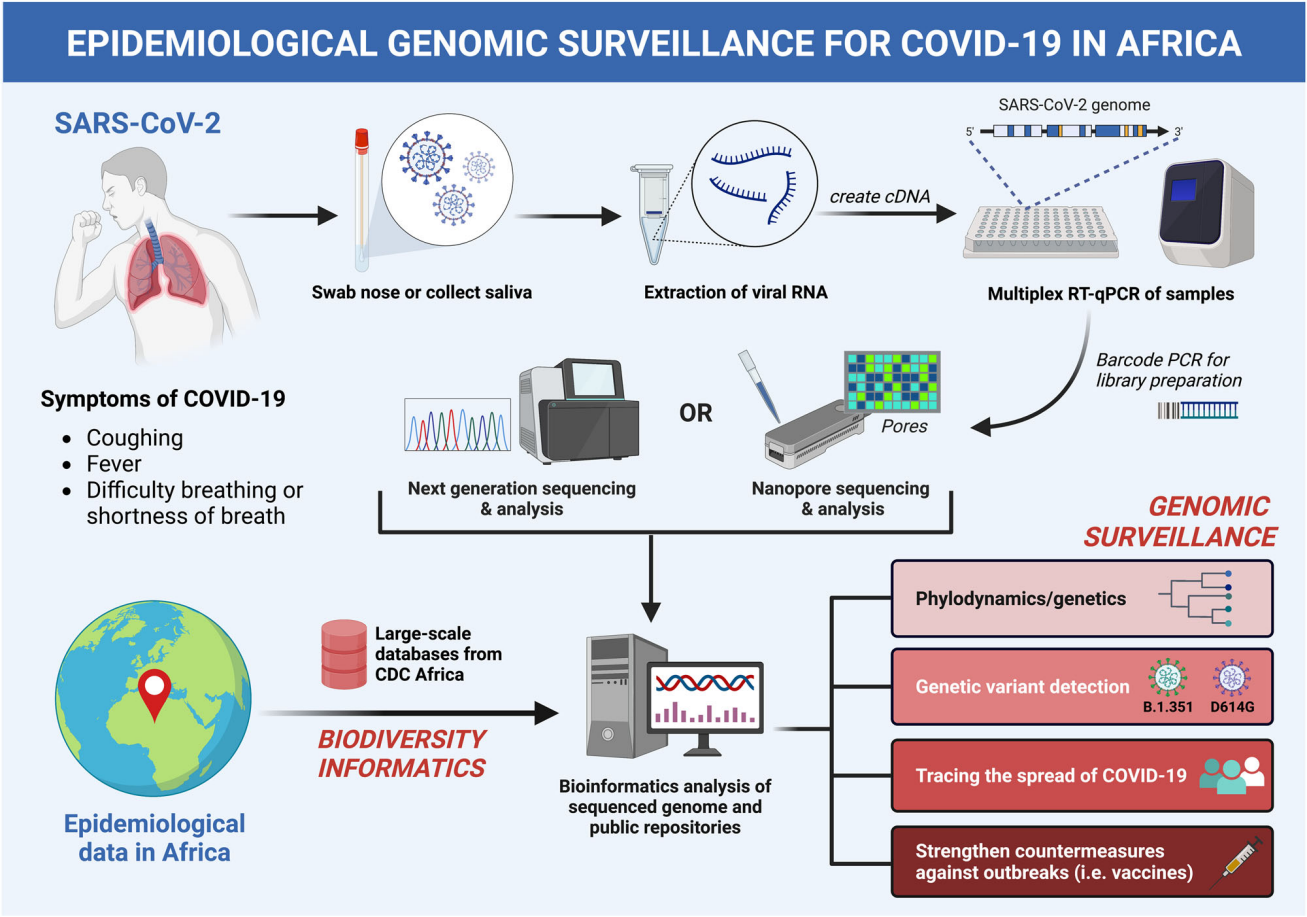
The process and outcomes of epidemiological genome surveillance from a SARS‐CoV‐2 infected patient and large‐scale databases from CDC Africa utilizing biodiversity informatics. (Original imaging created with biorender.com by H. H.). SARS‐CoV‐2, Severe Acute Respiratory Syndrome‐Coronavirus 2.

Within the modern era of medicine, genomic surveillance is an epidemiological application used to monitor genetic variants in pathogens and track the spread of pathogens from outbreaks to understand disease transmission (Figure 1). Genomic epidemiology in Africa is of growing interest due to the low costs of genome sequencing and the efficiency of processing large data sets of individual genomes to analyze viral variants and epidemic trends.^22^ Genomic surveillance was first adopted during one of the largest Ebola outbreaks in West Africa, where genome sequences of Ebola viruses were investigated to determine the transmission source.^23^ Given the strong evidence that the outbreak traced back to a similar variant causing the 2005 outbreak in Central Africa, the program established on‐site genome sequencing utilizing nanopore sequencing technology from remote locations and conducted swift bioinformatics analysis to determine genotypes of each variant.^23^ This research group was responsible for the first detection of Omicron variants.^23^ This was possible due to the ability of genomic surveillance to utilize phylogenetic approaches to detect viral lineages from patient samples. The concept of characterizing genetic variations by lineages was what brought important developments in influenza mitigation, antiviral development, and monitoring influenza resistance.^22^

The results were promising and showed that genomic surveillance could fill the gap that bridges contact tracing into effective COVID‐19 outbreak countermeasures. In the case of the Ebola virus, the genetic similarity of sequenced samples determined the outbreak to have originated from a natural source and spread via human‐to‐human transmission, thus eliciting the need to develop effective vaccines and create travel restrictions to prevent further transmission.^24^ In the case of COVID‐19, genomic surveillance has increased understanding of its genome and allowed the identification of possible variants.^25^ One of the first laboratories was implemented 3 days after the first case in Nigeria was announced and subsequently led to the establishment of the Network for Genomic Surveillance in South Africa (NGS‐SA) in March 2020.^26^ The organization uses sequencing data to trace SARS‐CoV‐2 viruses, to improve its diagnostic capabilities, and monitor new variants such as beta [B.1.351] and the D614G‐mutant virus.^27^ This is crucial since new evidence suggests that some concerning variations, such as B.1.351 and gamma, are not completely neutralized by vaccine‐induced antibodies.^23^ Increased use of these technologies may enable early detection of possible variations and fast adaptation of tactics to reduce outbreaks. Moreover, features of rapid data sharing from genomic surveillance can strengthen measures to contain outbreaks of COVID‐19 and allow for timely counter‐measures to genetic mutations accumulating in the virus as it spreads in Africa.

## 5 RESTRUCTURING BIODIVERSITY AND INFORMATICS FOR BETTER SUSTAINABILITY: FUTURE RECOMMENDATIONS AND PROSPECTS

Bioinformatics, a computerized process of handling and managing data on biodiversity, plays a paramount role in developing the necessary genomic tools and infrastructure to sustain operable epidemiological surveillance.^28^ Though existing institutions work hard to promote biodiversity informatics regionally, most African countries do not have policies that actively encourage regional collaboration and capacity building of bioinformatics.^29^ The lack of policies makes biodiversity informatics non‐existent due to challenges in aggregating and synthesizing large quantities of genome‐based data for effective surveillance. As a result, this could potentially blunt the benefits of genomic surveillance on pandemic preparedness and reduce investments in better technology.

In efforts to improve the sustainability of surveillance, the African Centres for Disease Control and Prevention Institute of Pathogen Genomics working group has implemented important factors in genomic surveillance, such as the mobilization of SARS‐CoV‐2 sequencing platforms, building a network of laboratories and bioinformatic experts to share expertize, and standardizing protocols to represent the data.^29^ Regardless of these developments, the lack of policies makes it difficult to aggregate and synthesize large quantities of genome‐based data for long‐term surveillance.^16^ This could potentially blunt the benefits of genomic surveillance on pandemic preparedness and reduce investments in better technology. Future governments should prioritize improving investments in informatic‐based policies that encourage the long‐term usage of genomic surveillance and promote the adoption of stronger biodiversity policies to national and governmental stakeholders.^17^, ^18^ This will aid in building adequate capacity to sustain long‐term genomic surveillance in Africa. It is also crucial to research the future uses of genetic surveillance for other viral outbreaks such as Monkeypox viruses and understand the socioeconomic circumstances of low‐middle‐income countries to gear global efforts in supporting the technologies necessary to appropriately manage the spread and develop mitigation based on the nations' needs.

## 6 CONCLUSION

Undoubtedly, Africa has made significant progress in COVID‐19 pandemic preparedness and has gained momentum in utilizing genomic surveillance for multiple outbreaks, from Ebola viruses to Influenza. However, the technological capacity needed to support genomic surveillance relies on policies regarding biodiversity informatics. Given the circumstance in Africa, the burden healthcare systems face from already strained COVID‐19‐related resources and overburdened healthcare workers must be taken into consideration when implementing sustainable and nationwide genomic surveillance. The rise in new variants and the gap in research on these mutations can hinder effective countermeasures against outbreaks and may contribute to the poor epidemiological tracing once seen during the Ebola epidemic. Therefore, countries should prioritize improving investments in informatic‐based policies that encourage the long‐term usage of genomic surveillance and promote the adoption of stronger biodiversity policies to national and governmental stakeholders. Future research should be dedicated to understanding the efficacy of implementing biodiversity informatics policies in the sustainability of wide‐scale genomic surveillance, disseminating possible risks of patient privacy in genomic data breaches, and uncovering effective measures against the rise in COVID‐19 variants through phylogenetic approaches.

## AUTHOR CONTRIBUTIONS

Abdullahi Tunde Aborode, Andrew Awuah Wireko, and Helen Huang conceptualized the topic, and coordinated reading, writing, and editing. Helen Huang, Aashna Mehta, Jacob Kalmanovich, Andrew Awuah Wireko, and Abdul‐Rahman Toufik contributed to reading, writing, and editing the original draft, and critical revision. All authors contributed to various aspects of reading, data collection, writing the original draft, and implementing changes for critical revision under the supervision of Abdullahi Tunde Aborode and Helen Huang.

## CONFLICT OF INTEREST

The authors declare no conflict of interest.

## Data Availability

No data available.
